# Diverse Genotypes of *Yersinia pestis* Caused Plague in Madagascar in 2007

**DOI:** 10.1371/journal.pntd.0003844

**Published:** 2015-06-12

**Authors:** Julia M. Riehm, Michaela Projahn, Amy J. Vogler, Minoaerisoa Rajerison, Genevieve Andersen, Carina M. Hall, Thomas Zimmermann, Rahelinirina Soanandrasana, Voahangy Andrianaivoarimanana, Reinhard K. Straubinger, Roxanne Nottingham, Paul Keim, David M. Wagner, Holger C. Scholz

**Affiliations:** 1 Central Institute of the Bundeswehr Medical Service, Munich, Germany; 2 Bundeswehr Institute of Microbiology & German Center for Infectious Diseases, Munich, Germany; 3 Center for Microbial Genetics and Genomics, Northern Arizona University, Flagstaff, Arizona, United States of America; 4 Institut Pasteur de Madagascar, Antananarivo, Madagascar; 5 Institute for Infectious Medicine and Zoonoses, Munich, Germany; 6 Translational Genomics Research Institute North, Flagstaff, Arizona, United States of America; Beijing Institute of Microbiology and Epidemiology, CHINA

## Abstract

**Background:**

*Yersinia pestis* is the causative agent of human plague and is endemic in various African, Asian and American countries. In Madagascar, the disease represents a significant public health problem with hundreds of human cases a year. Unfortunately, poor infrastructure makes outbreak investigations challenging.

**Methodology/Principal Findings:**

DNA was extracted directly from 93 clinical samples from patients with a clinical diagnosis of plague in Madagascar in 2007. The extracted DNAs were then genotyped using three molecular genotyping methods, including, single nucleotide polymorphism (SNP) typing, multi-locus variable-number tandem repeat analysis (MLVA), and Clustered Regularly Interspaced Short Palindromic Repeats (CRISPR) analysis. These methods provided increasing resolution, respectively. The results of these analyses revealed that, in 2007, ten molecular groups, two newly described here and eight previously identified, were responsible for causing human plague in geographically distinct areas of Madagascar.

**Conclusions/Significance:**

Plague in Madagascar is caused by numerous distinct types of *Y*. *pestis*. Genotyping method choice should be based upon the discriminatory power needed, expense, and available data for any desired comparisons. We conclude that genotyping should be a standard tool used in epidemiological investigations of plague outbreaks.

## Author Summary

*Yersinia pestis* is a highly pathogenic bacterium and the causative agent of human plague. It has caused three recognized pandemics and is a current human health problem in many countries of Africa, Asia and the Americas, including Madagascar. The pathogen cannot be eradicated from natural plague foci as it persists in various known and cryptic rodent reservoir species. Genotyping is a critical tool in understanding the molecular epidemiology and possible kinetics of plague. In the present study, we succeeded in extracting DNA and genotyping directly from human clinical samples from Madagascar. We applied three different methods, including single nucleotide polymorphism (SNP) typing, multi-locus variable-number tandem repeat (VNTR) analysis (MLVA), and Clustered Regularly Interspaced Short Palindromic Repeats (CRISPR) analysis. Relative to their discriminatory power, all three methods provided important genotype information useful for understanding the molecular epidemiology of the disease, revealing that multiple, distinct genotypes caused human plague in Madagascar within one year, 2007.

## Introduction

*Yersinia pestis*, the causative agent of plague, is one of the most deadly zoonotic pathogens on record, with hundreds of millions of human deaths attributed to it over the course of three historical pandemics [1]. Human cases typically present in one of three forms, including bubonic, septicemic, and the contagious pneumonic form [1], and are notifiable to the World Health Organization [2,3]. Since its registration as a notifiable disease in 1954, plague has been reported in various Asian, American and African countries [2,3]. Plague persists in these countries in various known and other cryptic rodent reservoir species in multiple established foci [1,4,5]. Understanding the epidemiology of this pathogen in these natural reservoirs and in human outbreaks requires the development and implementation of effective molecular genotyping tools that can successfully identify and characterize *Y*. *pestis*, preferably from a wide variety of sample types.

Plague was first introduced to Madagascar in 1898 during the third pandemic. The disease then spread to the capital city of Antananarivo in 1921 and became established in the surrounding highlands while disappearing from the coastal areas [6,7]. Plague currently persists in two large foci above 800 m in elevation in the central and northern highlands. It remains a significant public health concern, with hundreds of human cases reported annually [7]. Malagasy plague cases are categorized as confirmed (isolation of *Y*. *pestis*), presumptive (positive by microscopy but no strain isolation), or suspected (negative test results or no tests performed, but clinical symptoms). Due to logistical difficulties, the frequency of biological case confirmation (confirmed and presumptive cases) is very low in Madagascar (21.4% of suspected cases from 1957–2001) [8], although this can be increased using F1 antigen detection [9]. Historically, genotyping of *Y*. *pestis* in Madagascar has been limited to confirmed cases [10–12]. However, recently developed molecular assays provide the opportunity to directly investigate clinical samples without the need for strain isolation.

In the present study, we investigated clinical samples by extracting DNA and then using three different genotyping methods. Each method could successfully be applied despite the background of human DNA and revealed important genotype information useful for understanding the molecular epidemiology of *Y*. *pestis* in Madagascar.

## Methods

### Ethics statement

Samples were de-linked from the originating patients and analyzed anonymously. All adult subjects provided informed consent, and a parent or guardian of any child participant provided informed consent on their behalf. The consent was approved by signature. Data collection and investigation on human samples were finally approved by the Ethical Committee of the Ministry of Health of Madagascar.

### Sample collection

In 2007, 99 human clinical samples were collected from 21 districts in Madagascar (S1 Table). They originated from suspected and confirmed bubonic and pneumonic human plague cases. Tested clinical material included bubo aspirates or sputum collected by the Malagasy Central Laboratory for plague and the Institut Pasteur de Madagascar (provided by Lila Rahalison). All 99 cases from which the clinical specimens were collected were F1 antigen positive and 93 were culture positive (S1 Table). DNA was extracted from inactivated clinical samples using the QIAamp DNA Mini Kit (Qiagen, Hilden, Germany).

### Genotyping

DNAs were screened, as previously described, across assorted SNPs—Mad-08 through Mad-49 from reference [12] and Mad-57 through Mad-78 from reference [11]—in a hierarchical fashion. Specifically, SNP Mad-43 was screened first to determine whether a sample belonged in Group I or II, two previously described major groups in Madagascar [12,13]. Then, additional Group I or II SNPs were screened to determine what SNP-determined group (i.e., node) each sample belonged in. Sequencing was performed to determine SNP states for some samples that yielded ambiguous results using the melt mismatch amplification mutation assays (Melt-MAMA) (contact corresponding author for specific methods). Further genotyping was attempted on all 99 samples using a 43-locus MLVA system [14] and by sequencing three CRISPR loci [15–18], as previously described.

### Phylogenetic and geographic analyses

**Fig 1:**
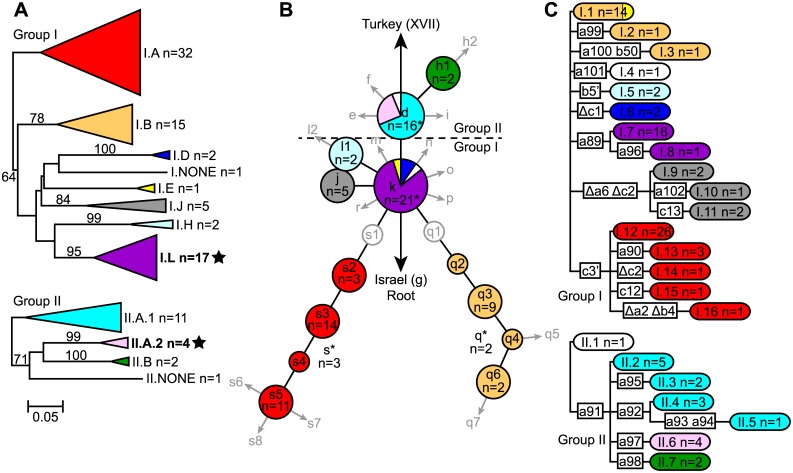
MLVA (A), SNP (B), and CRISPR (C) based phylogenies of 93 *Y*. *pestis* clinical samples from Madagascar in 2007. MLVA subclades were named as in reference [12] for the 8 of 15 previously described subclades identified here, except for subclade II.A, which was subdivided into subclades II.A.1 and II.A.2 in this analysis. Two new subclades described here, including II.A.2, are bolded and marked with stars. One Group I sample and one Group II sample did not fall into any recognized MLVA subclade and so were classified as I.NONE and II.NONE, respectively (A). SNP phylogeny nodes were named as in references [11–13] and include 13 of the 31 nodes described there. Previously described nodes that were not represented in this study are indicated by gray circles and arrows in the SNP phylogeny (B). The numbers of samples in each MLVA subclade and SNP phylogeny node with >1 sample as well as the total number of samples in each CRISPR genotype are indicated. Asterisks indicate 7 samples with incomplete SNP data that were assigned to a SNP phylogeny lineage or node based upon MLVA data (B). Color shading in each phylogeny is based upon the identified MLVA subclade and corresponds to the MLVA subclade colors in reference [12] for the 8 previously described MLVA subclades. Bootstrap values ≥50 supporting MLVA phylogeny branches (A) and specific CRISPR mutations marking CRISPR phylogeny branches (C) are also indicated. Note that CRISPR genotypes I.1 and II.1 are identical and consist of the following CRISPR pattern: a1-a2-a3-a4-a5-a6-a7-a8 b1-b2-b3-b4-b5 c1-c2-c3 (C). An updated CRISPR dictionary is available in S2 Table.

**Fig 2:**
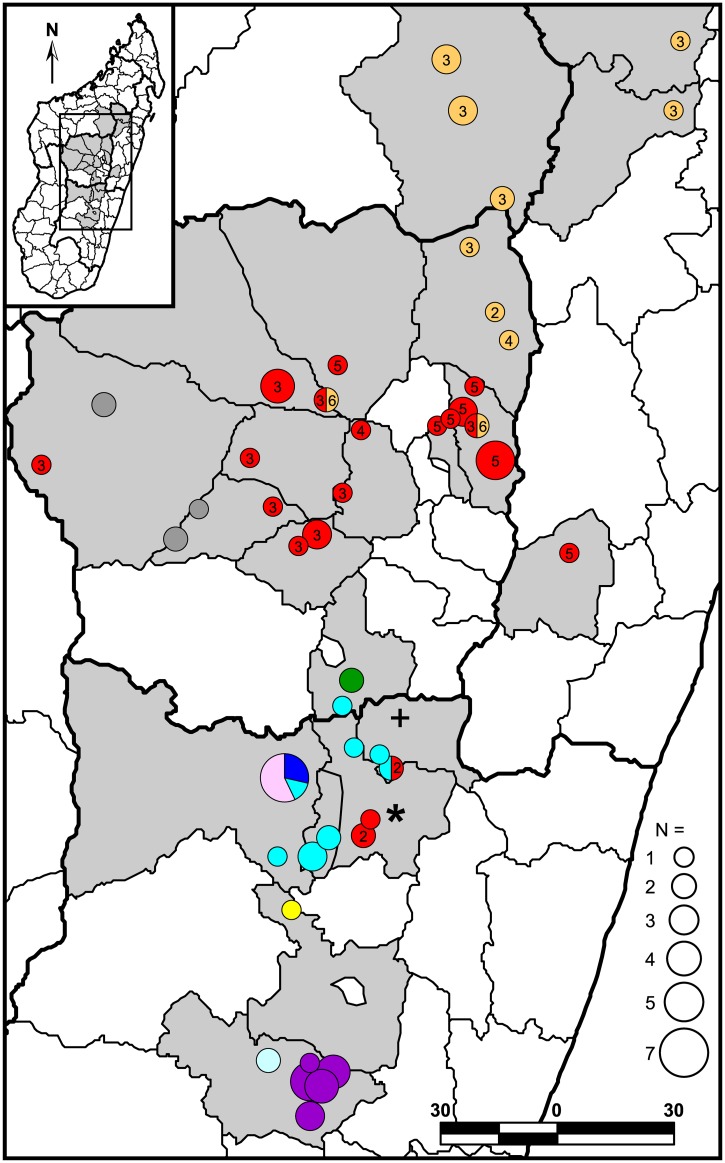
Geographic distribution of 93 Malagasy *Y*. *pestis* clinical samples from 2007. Light gray shaded polygons indicate Madagascar districts where *Y*. *pestis* samples used in this study were obtained (S1 Table, column E,F). Circles represent the locations of the communes where samples were collected. Colors within the mapped circles correspond to the MLVA subclade color designations in Fig 1. Divisions within circles indicate that multiple MLVA subclades were found at that location. Numbers within MLVA subclade I.A (red) and I.B (orange) circles and pie chart slices indicate the node within lineages s and q, respectively, of the SNP phylogeny in Fig 1 that the mapped samples belong to. Where the SNP genotype could not be definitively determined for any of the subclade I.A or I.B samples from a particular location, no number is shown. Unaffiliated Group I and II samples (i.e., subclades I.NONE and II.NONE) are indicated by a “*” and a “+,” respectively.

Clinical samples that were successfully genotyped using MLVA were analyzed in conjunction with data from 262 previously published samples in a neighbor-joining analysis to determine MLVA subclades [12]. Phylogenies were then constructed using the SNP, MLVA, and CRISPR data for the successfully genotyped clinical samples, with separate phylogenies generated for Groups I and II (as determined by SNP data) for the MLVA and CRISPR based phylogenies (Fig 1 and S1 Table). The MLVA based phylogenies were generated in MEGA6 [19] as neighbor-joining dendrograms using mean character based distance matrices and include bootstrap values ≥50 generated in PAUP 4.0b10 (D. Swofford, Sinauer Associates, Inc., Sunderland, MA) based upon 1,000 simulations. The geographic distributions of the identified MLVA subclades were mapped using ArcGIS 10.2.1 for Desktop (ESRI, Redlands, CA) (Fig 2).

## Results

SNP, MLVA, and CRISPR typing all provided robust genotyping overall, despite being used on DNA extracted from clinical samples that included serous, bloody, mucous, or putrified materials. Only four samples amplified very poorly in the SNP and MLVA assays and so were not included in the phylogenetic or geographic analyses. These failures appeared unrelated to culturing success (S1 Table). Two other samples displayed mixed genotypes when analyzed using MLVA and so were also excluded, leaving 93 samples in the phylogenetic and geographic analyses (S1 Table).

The MLVA, SNP, and CRISPR phylogenies demonstrated remarkable congruence. Ten MLVA subclades were identified, eight of which corresponded to previously described subclades [12] and two of which were new (Fig 1 and S1 Table). The ten identified subclades were found in geographically distinct areas (Fig 2) and most corresponded to a SNP phylogeny node/lineage and/or to a CRISPR group (Fig 1 and S1 Table). Although all three methods were able to identify major groups, they differed in their discriminatory power and general applicability to clinical samples. MLVA provided the greatest discriminatory power with 81 unique MLVA genotypes among the 93 samples. However, this method was expensive to run (ten multiplexed PCR reactions and six capillary electrophoresis runs per sample [14]) and was not successful for 4 of the 99 samples (S1 Table). In contrast, there were 22 CRISPR and 13 SNP genotypes among the 93 samples (Fig 1). CRISPR required only three sequencing runs and was successful for all 99 samples, identifying 17 new and, to our knowledge, Madagascar-specific CRISPR spacers. These new spacers were named in accordance with previously published *Y*. *pestis* CRISPR spacers [15,16,18] (S2 Table), with new consecutive numbers assigned to new spacers within each of the three loci. This is in contrast to the CRISPR naming strategy recently published for *Y*. *pseudotuberculosis*, in which CRISPR spacers were assigned consecutive numbers without regard to the specific locus [20]. The Melt-MAMA assays predominantly used to genotype the SNPs were very economical but demonstrated a higher failure/ambiguous call rate than either MLVA or CRISPR on these clinical samples (S1 Table).

## Discussion

Multiple genotypes continue to cause human plague in Madagascar. Within this single plague season, a total of ten MLVA subclades were identified to cause disease, eight of which were previously described (Fig 1). These subclades showed geographic distributions consistent with earlier observations (Fig 2) [12]. The geographic distributions of the SNP genotypes in lineages q and s (i.e., MLVA subclades I.B and I.A, respectively) were similarly compatible with previous reports [11,12]. Some expansions in the known geographic distributions of several subclades were observed in this study, the most significant of which involved the observation of several subclade I.B samples in three additional northeastern central highlands districts (Fig 2). Whether these are true expansions or if this is due to a lack of samples from these geographic areas in the previous study is unknown. Of seven other previously described MLVA subclades not seen here, two were speculated to be currently extinct (I.I and I.K) and three were restricted to geographic areas not sampled in this study (I.C and I.G in the northern highlands and II.C in the Betafo district) [12]. The failure to observe the remaining two previously described subclades (I.F and II.D), despite sampling within their known geographic distributions, could be due to chance or may indicate the extinction of these two subclades as of 2007.

In addition to the above, two new MLVA subclades were also identified (Fig 1). Subclade II.A.2 appears to be a previously unrecognized subdivision within the previously identified subclade II.A based upon a neighbor-joining analysis of the 93 samples from this study and 262 previously published samples [Fig 2, S1 Table in 12]. Subclade II.A was not statistically supported in the previous analysis and so may not be a robust group [12]. Subclade I.L appears entirely new and was geographically restricted to district Ambalavao in the southern central highlands (Fig 2). This subclade may be newly emerged or may not have been previously observed due to the very low sampling in this district in the previous study [12].

Using CRISPR, a total of 18 new and, apparently, Madagascar-specific spacers have been identified, 17 here and one previously [17]. Of these, 13 belong to the A locus, two to the B locus, and three to the C locus (S2 Table). Compared to a total of 140 *Y*. *pestis* specific spacers published worldwide [15,16,18], this is a fairly high number for a geographic area the size of Madagascar, although it is consistent with the high plague activity in this endemic country and the similarly large numbers of SNP and MLVA genotypes that have been reported from Madagascar [11–13].

Of the CRISPR genotypes observed in Madagascar, the a1-a2-a3-a4-a5-a6-a7-a8 b1-b2-b3-b4-b5 c1-c2-c3 CRISPR genotype may represent the root CRISPR genotype in Madagascar, as it was shared by samples in Groups I (CRISPR genotype I.1, n = 14) and II (CRISPR genotype II.1, n = 1) (Fig 1 and S1 Table). This CRISPR genotype was also observed in the majority of the genotyped samples in a recent study of a 2011 pneumonic plague outbreak that occurred outside the recognized northern highlands plague focus in northern Madagascar. These samples all belonged to SNP node k [17], placing them in CRISPR genotype I.1 described here. Two other genotyped samples from that study, one from a rat trapped in one of the outbreak areas, commune Ambarakaraka, and one from a reference sample from the northern Bealanana district, possessed another CRISPR genotype seen here, genotype I.15 [17] (Fig 1 and S1 Table). This is interesting, given the association of this CRISPR genotype with MLVA subclade I.A (Fig 1), as this is the only indication of this MLVA subclade in northern Madagascar. This hypothesis could be confirmed by genotyping these samples with either SNP Mad-57, marking the s lineage [11], or with MLVA, but is strongly suggested by the congruence observed here between MLVA and CRISPR (Fig 1). The spread of MLVA subclade I.A to northern Madagascar would not be unexpected, given the previous success of this genotype in spreading throughout the central highlands and to the port city of Mahajanga [11,12]. Previously published SNP and MLVA data for samples from northern Madagascar are limited [11,12] and a more extensive analysis using a larger set of samples from this region could determine the timing and extent of the spread of this MLVA subclade to northern Madagascar.

In this clinical sample analysis, SNPs provided less discriminatory power than MLVA or CRISPR and the Melt-MAMA SNP genotyping assays demonstrated a higher failure/ambiguous call rate. However, additional whole genome sequencing and SNP discovery, particularly of strains from the d or k nodes, will likely lead to additional SNP genotypes and could potentially allow identification of all currently described MLVA subclades in Madagascar. Also, SNP analysis remains the only reliable method for accurately differentiating between Groups I and II `^12^] (Fig 1). In addition, the failure/ambiguous call rate could likely be improved using a different SNP genotyping method and, given the hierarchical nature of the SNP analysis, could still be relatively economical even if a more expensive method were used.

## Conclusions

The MLVA, SNP, and CRISPR results reported here indicate that direct DNA extraction and genotyping of clinical samples is possible using these methods and may be used for epidemiological investigations, sidestepping the need for obtaining bacterial cultures. Method choice should be based upon the discriminatory power needed, expense, and available data for any desired comparisons. Multiple genotypes continue to be responsible for causing human plague in Madagascar and continue to be observed in geographically distinct areas.

## Supporting Information

S1 Table Clinical samples in this study. (XLSX)

S2 Table Updated CRISPR dictionary. (XLS)
